# Use of contraception during first sexual intercourse among Norwegian adolescents: a national cross-sectional study

**DOI:** 10.1186/s12889-024-19009-4

**Published:** 2024-06-06

**Authors:** Live Solveig Nordhagen, Hilde Egge, Marja Leonhardt

**Affiliations:** 1 VID Specialized University, Oslo, Norway; 2 Norwegian National Advisory Unit on Concurrent Substance Abuse and Mental Health Disorders, Innlandet Hospital Trust, Elverum-Hamar, Norway

**Keywords:** Contraception, First sexual intercourse, Adolescent, Alcohol intoxication, Narcotic substance use

## Abstract

**Background:**

Most Norwegian adolescents experience their first sexual intercourse during late adolescence. Use of contraception is important to avoid unwanted pregnancy, while condoms can also protect against sexually transmitted diseases. There are few studies on the use of contraception at first sexual intercourse, most with varying results, and some studies have only examined the use of contraception among girls. In our study, we aimed to determine the use of contraception at first sexual intercourse, and to investigate associations between use of contraceptives at first sexual intercourse, sociodemographic factors, and alcohol and other substance use.

**Methods:**

The study was based on data from the national electronic youth survey Ungdata, conducted in 2020–2022 among 113 049 upper secondary pupils (15–19 years) in Norway, which was around 65% of pupils attending upper secondary school during the study period. Descriptive analysis was used to estimate the prevalence of contraceptive use at first sexual intercourse, and multivariate logistic regression analyses to investigate the association between contraceptive use, sociodemographic factors, and alcohol intoxication and substance use.

**Result:**

32% of Norwegian adolescents did not use contraception at first sexual intercourse. More girls (57.4%) than boys (42.6%) reported use of contraception. Factors associated with non-use of contraception during first sexual intercourse among boys were having parents with no college /university education (OR = 1.22: CI 1.13–1.32), perceived poor family finances (OR = 1.22: CI 1.06–1.40), alcohol intoxication, and use of cannabis or other narcotic substances during the past 12 months. The same factors were associated with non-use of contraception among girls. Additionally, being older than 16 years (OR = 1.13: CI 1.06–1.19) was also associated with non-use of contraception at first sexual intercourse.

**Conclusion:**

Many adolescents did not use contraception at first sexual intercourse. Alcohol intoxication and use of cannabis or other narcotic substances were associated with a lower likelihood of using contraceptives. This highlights the importance of preventive efforts including earlier prevention education that focuses more on the consequences of not using contraception in order to prevent unwanted pregnancies and sexually transmitted infections.

## Background

Use of contraception is important to avoid unwanted pregnancy, and condoms additionally protect against sexually transmitted diseases. Over the past years, there has been an increase in pregnancy termination in Norwegian girls in the age group 15 to 19 years, to 5.9 per 1000 [1]. According to a Norwegian report from 2022, there has been an increase in sexually transmitted diseases such as chlamydia infections (people under 25 years old accounted for 67% of all cases), and gonorrhoea, particularly among young heterosexuals [2]. It is therefore crucial to provide easy access to contraceptives and promote health literacy about various contraceptives to prevent unwanted pregnancies and sexually transmitted diseases [3, 4].

Most adolescents experience their first sexual intercourse during late adolescence, and the age of sexual debut for Norwegian girls and boys has been stable over the past decades. The sexual debut age is around 17.7 years for girls and 18.6 years for boys in Norway [5, 6], which is similar to other Nordic countries [7]. There are few studies on adolescent use of contraceptives at first intercourse and they show varying results. One Norwegian study from the period 2017–2019 showed that 34% of adolescents did not use contraception at sexual debut [8], while a study from 2020 found that 17.4% of boys and 10.4% of girls did not use contraception at first sexual intercourse [9]. A Scandinavian study from 2011 to 2012 found that 14.1% of Norwegian, 16.6% of Swedish, and 9.6% of Danish girls did not use contraception at sexual debut [10]. A national survey from the United States conducted in 2011–2017 concluded that 21% of girls did not use contraception during their first sexual intercourse [11]. A study conducted in 2009–2010 in Norway and Croatia showed that using a condom during first sexual intercourse was associated with use of contraception during later sexual activity among Norwegian and Croatian girls and Croatian boys, but not in Norwegian boys [12]. Older studies from Denmark (2004) and the USA (2002) found that females who did not use contraception at first sexual intercourse were less likely to use contraception at later intercourse and more often had induced abortion [12–14].

Several studies have examined factors that may be associated with lack of contraceptive use by adolescents. Research shows that drinking alcohol [8, 15], smoking [10], and use of cannabis [8, 15] and other drugs [15] are associated with not using contraception. The past ten years have seen a change in alcohol and cannabis use among high school students. There is a declining trend in alcohol use in Western Europe and North America [16]. The use of cannabis has increased in recent years, and cannabis is the most widely used illegal drug in Europe [16–19]. The use of other narcotic substances such as cocaine is lower but has increased in recent years [17].

The availability of contraception for adolescents in Norway has improved in recent years; public health nurses and midwives can both prescribe contraceptives for adolescents. Public health nurses and midwives are entitled to request contraception from 12 years, the requests being made after an assessment by a health professional and on indication [20]. Many Norwegian municipalities have free youth health centres, which are open after school hours and provide contraception advice and ensure accessible and safe contraception. Most upper secondary schools also have their own public health nurse who provides sex education and is available for counselling, which is provided for in school curricula [21].

Given the lack of recent studies and inconsistent findings on contraceptive use in adolescents, along with increases in substance use, induced abortion and sexually transmitted diseases in Norwegian adolescents, it is crucial to gain insights into their contraceptive practices in order to implement focused preventive measures. This study therefore aims to examine the use of contraception during first sexual encounters and explore the potential associations between this practice and socio-demographic factors, alcohol intoxication and substance use.

## Method

### Design and procedure

**Fig. 1:**
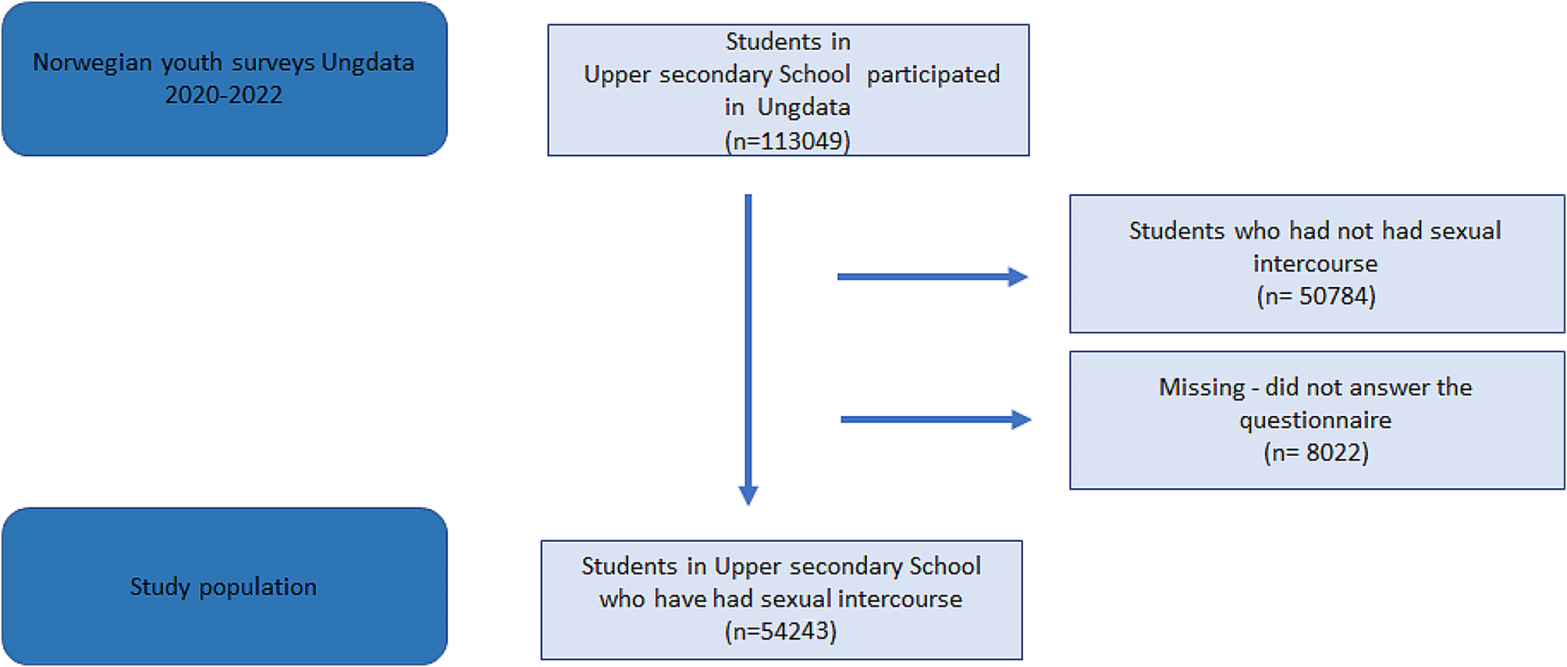
Flow diagram visualizing the selection of the study population

This study is based on data from the Norwegian youth survey Ungdata, an ongoing national web-based questionnaire survey of students in secondary school from all over the country. The Ungdata surveys are conducted in most of the Norwegian municipalities, typically every third year. They are administered by NOVA Norwegian Social Research at Oslo Metropolitan University in collaboration with the KoRus Regional Centres of Expertise on Alcohol and Drugs. The purpose of Ungdata is to investigate adolescents’ health, well-being, quality of life, relationships with friends and parents, school and future plans, local community, leisure activities, substance use, and sexual behaviour (see https://www.ungdata.no/english/). Participants and their parents were informed about the survey by letter in advance. Parents of pupils under 16 had to consent to their child’s participation in Ungdata. Upper secondary school pupils of 16 years or above could provide consent themselves. Parents of pupils under the age of 18 still had the possibility to refuse to allow their child to take part in the survey. Participation was voluntary and was conducted during school hours with a teacher or a public health nurse present. The first part of the survey is used in all participating municipalities whereas the second part comprises questions that can be chosen by each municipality. Therefore, the number of responses varies greatly between the mandatory and elective parts of the questionnaire. In this study, we used self-reported data from the surveys conducted in 2020–2022 of pupils in grades 1, 2 and 3 of upper secondary school. A total of 113 049 adolescents participated, which is 68%, 67% and 65% of the proportion of adolescents attending upper secondary school during the same period [22–24]. The respondents were asked “Have you ever had sexual intercourse with anyone?”. Adolescents who had not had sexual intercourse were excluded. Thus, the population used for the present analysis consisted of 54 234 adolescents, see Fig. 1.

## Measures

### Outcome variable

The use of contraception at first sexual intercourse was assessed through the question “Did you use contraception when you had sexual intercourse for the first time?” with the possible response options “Yes”, “No” and “Unsure/don’t remember”. The variable was dichotomized for the purpose of logistic regression and the option “Unsure/don’t remember” (2215 boys and 1691 girls, 7.5% of the study population) was considered as missing data.

#### Independent variables

The sociodemographic factors comprised the variables of school grade, gender, parental education, and perceived family finances. School grade was used as a proxy for age. Perceived family finances were measured by the question “Has your family had a good or bad financial situation in the past two years?” with the five response options “well off all the time”, “mostly well off”, “neither well off nor badly off”, “mostly badly off” and “badly off all the time”, which were recoded into “perceived good family finances”, “perceived neither good nor poor family finances”, and “perceived poor family finances” based on other studies using data from the Ungdata surveys [25]. Education of parents/guardians was based on the question “Do your parents/guardians have a university or college education?” with the response options “No, neither of them”, “Yes, one of them”, “Yes, both”. This variable was dichotomized to “Higher education” and “No higher education”. The variable age at first intercourse was dichotomized to ≤ 15 years and ≥ 16 years, as 16 years is the age of sexual consent in Norway [26]. Alcohol intoxication, cannabis use and the use of other narcotic substances were assessed by asking “How many times have you done any of these things in the last year (last 12 months): a) drunk so much alcohol that I was intoxicated, b) used cannabis, and c) used other narcotic substances”, with the response options “not at all”, “once”, “2–5 times”, “6–10 times” and “11 or more times”. For the purpose of analysis, we recoded these variables into “no episodes”, “1–5 episodes” and “6 or more episodes”.

## Statistical analysis

The characteristics of the study sample were described by frequencies and percentages. Comparisons between use and non-use of contraception were made using the χ2 test for categorical data. Bi- and multivariate logistic regression analysis were used to examine the relationship between non-use of contraception and the independent variables. All logistic regression analysis was stratified by gender. The first category of the independent variables was set as reference to simplify interpretation. We controlled the logistic regression analyses for possible confounders such as sociodemographic factors and alcohol and substance use. Results are expressed as odds ratios (OR) with a 95% confidence interval (CI). Statistical significance was set to *p* < 0.05. All analyses were performed in IBM SPSS Statistics for Windows®, Version 28.

## Ethics

Data were obtained from an established dataset. The schools that participated in Ungdata were required to provide their pupils with necessary information about the purpose of the survey, explain that participation was voluntary and mention other privacy issues prior to the survey. All parents were also sent an advance letter containing information about Ungdata. It was pointed out that the dataset did not contain personal information. NOVA Norwegian Social Research had overall responsibility for ensuring that data were collected in accordance with applicable laws, regulations and research ethics guidelines. The Regional Ethics Committee considered that the project fell outside the provisions of the Norwegian Health Research Act and that the use of passive consent was appropriate. Therefore, the data protection office of Inland Hospital Trust (reference number 18,778,329) approved this analysis.

## Results

### Characteristics of the study population

Table 1 describes the study population, consisting of 29 154 (54.6%) girls and 24 196 (45.4%) boys. Age at first sexual intercourse was ≤ 15 years for 49.3% (26 182) and ≥ 16 years for 50.7% (26 928) of the participants. Most of the pupils had parents/guardians with college/university education and perceived their family finances as good. There was a higher proportion of boys reporting non-use of contraception (48.1%) than use of contraception (42.6%) at first sexual intercourse. Those adolescents who had been intoxicated with alcohol six or more times, or used cannabis or other narcotic substances more than once, during the past 12 months were more likely to report not using contraception. The characteristics of the respondents are presented in Table 1.

**Table 1:** Characteristics of the study population by use and non-use of contraception at first sexual intercourse

		Total *n* = 54,243	Use of contraception *n* = 33,865 (68.3%)	Non-use of contraception *n* = 15,706 (31.7%)	
	*n*	*n*%	*n*%	*n*%	*P*-value
**Gender**	53 350				< 0.001*
Boy		24 196 (45.4)	14 244 (42.6)	7 381 (48.1)	
Girl		29 154 (54.6)	19 226 (57.4)	7 977 (51.9)	
**Grade levels in upper secondary school**	53 893				0.004*
1st grade		18 293 (33.9)	11 442 (34.0)	5 092 (32.6)	
2nd grade		20 452 (37.9)	12 718 (37.8)	5 944 (38.1)	
3rd grade		15 148 (28.1)	9 486 (28.2)	4 575 (29.3)	
**Age at first sexual intercourse**	53 110				0.004*
≤ 15 years		26 182 (49.3)	16 129 (48.2)	7 697 (49.6)	
≥ 16 years		26 928 (50.7)	17 342 (51.8)	7 821 (50.4)	
**Education of parents/guardians**	52 714				< 0.001*
College/university (one or both)		42 030 (79.7)	26 700 (80.8)	12 002 (78.6)	
No college/university		10 684 (20.3)	6 342 (19.2)	3 273 (21.4)	
**Perceived family finances**	53 547				< 0.001*
Good finances		41 271 (77.1)	26 246 (78.2)	11 691 (75.5)	
Neither good nor poor finances		9 238 (17.3)	5 625 (16.8)	2 733 (17.6)	
Poor finances		3 038 (5.7)	1 682 (5.0)	1 062 (6.9)	
**Alcohol intoxication (last 12 months)**	52 363				< 0.001*
No episodes		10 342 (19.8)	7 203 (21.8)	2 140 (14.2)	
1–5 episodes		18 013 (34.4)	11 911(36.1)	4 706 (31.2)	
6 or more episodes		24 008 (45.8)	13 887 (42.1)	8 229 (54.6)	
**Cannabis use (last 12 months**)	52 139				< 0.001*
No episodes		40 718 (78.1)	26 849 (81.6)	10 620 (70.8)	
1–5 episodes		7 642 (14.7)	4 254 (12.9)	2 765 (18.4)	
6 or more episodes		3 779 (7.2)	1 783 (5.4)	1 605 (10.7)	
**Other narcotic substances (last 12 months)**	52 090				< 0.001*
No episodes		47 373 (90.9)	30 671 (93.4)	12 934 (86.3)	
1–5 episodes		3 301 (6.3)	1 617 (4.9)	1 368 (9.1)	
6 or more episodes		1 416 (2.7)	554 (1.7)	688 (4.6)	

*All statistically significant relationships (*p*-value < 0.05)

### Associations between non-use of contraception at first sexual intercourse, sociodemographic factors, alcohol intoxication and substance use

The results of the logistic regression models are presented in Table 2. In the unadjusted analysis, a notable link was observed between non-use of contraception during the initial sexual encounter and all independent variables, except for grade levels in upper secondary school among girls, age of 16 years or above at first sexual intercourse, and boys with perceived neither good nor poor family finances. Factors associated with non-use of contraception during first sexual intercourse among boys were having parents without college/university education (OR = 1.22; CI 1.13–1.32), perceived poor family finances (OR = 1.22; CI 1.06–1.40), alcohol intoxication 1–5 times (OR = 1.24; CI 1.13–1.36) and six or more times (OR = 1.86; CI 1.70–2.03) during the last 12 months, cannabis use 1–5 times (OR = 1.15; CI 1.06–1.26) and six or more times (OR = 1.25; CI 1.10–1.41) during the last 12 months, and using other narcotic substances 1–5 times (OR = 1.44; CI 1.28–1.62) and six or more times (OR = 1.85; CI 1.55–2.21) during the last 12 months. Factors associated with non-use of contraception among girls were age > 16 years (OR = 1.13; CI 1.06–1.19), having parents without college/university education (OR = 1.08; CI 1.01–1.16), perceived poor family finances (OR = 1.15; CI 1.02–1.29), alcohol intoxication 1–5 times (OR = 1.37; CI 1.26–1.50) and six or more times (OR = 1.74; CI 1.60–1.90) during the last 12 month, having used cannabis 1–5 times (OR = 1.42; CI 1.31–1.54) and six or more times (OR = 1.52; CI 1.32–1.76 ) during the last 12 months and having used other narcotic substances 1–5 times (OR = 1.40; CI 1.23–1.59) and six or more times (OR = 1.67; CI 1.33–2.09) during the last 12 months.

**Table 2:** Associations between the independent variables and non-use of contraception at first sexual intercourse (*n* = 54 243)

	Crude OR (95% CI)		Adjusted OR (95% CI)	
	**Girls** (*n* = 29 154)	**Boys** (*n* = 24 196)	**Girls** (*n* = 29 154)	**Boys** (*n* = 24 196)
**Grade levels in upper secondary school**				
1st grade (ref)				
2nd grade	0.954 (0.895–1.016)	1.177 (1.101–1.257) *	9.893 (0.835–0.956)	1.112 (1.035–1.195) *
3rd grade	0.974 (0.912–1.040)	1.309 (1.216–1.409) *	0.863 (0.803–0.928)	1.169 (1.076–1.269) *
**Age at first sexual intercourse**				
≤ 15 years(ref)				
≥ 16 years	1.010 (0.958–1.064) *	0.897 (0.847–0.949)	1.128 (1.065–1.194) *	0.969 (0.910–1.031)
**Education of parents/guardians**				
College/university (one or both) (ref)				
No college/university	1.089 (1.020–1.162) *	1.202 (1.120–1.291) *	1.083 (1.010–1.161) *	1.217 (1.126–1.316) *
**Perceived family finances**				
Good finances (ref) Neither good nor poor finances	1.133 (1.059–2.212) *	1.060 (0.980–1.147)	1.083 (1.008–1.164) *	1.012 (0.929–1.102)
Poor finances	1.335 (1.198–1.487) *	1.414 (1.246–1.604) *	1.149 (1.022–1.292) *	1.220 (1.061–1.403) *
**Alcohol intoxication (last 12 months)**				
No episodes (ref)				
1–5 episodes	1.395 (1.283–1.517) *	1.342 (1.231–1.462) *	1.371 (1.256–1.497) *	1.241 (1.133–1.358) *
6 or more episodes	1.902 (1.755–2.061) *	2.210 (2.042–2.392) *	1.742 (1.597–1.901) *	1.861 (1.704–2.033) *
**Cannabis use (last 12 months)**				
No episodes (ref)				
1–5 episodes	1.732 (1.609–1.864) *	1.518 (1.405–1.640) *	1.421 (1.311–1.540) *	1.152 (1.057–1.257) *
6 or more episodes	2.205 (1.955–2.487) *	2.102 (1.918–2.305) *	1.521 (1.317–1.757) *	1.246 (1.103–1.407) *
**Use of other narcotic substances (last 12 months)**				
No episodes (ref)				
1–5 episodes	1.972 (1.761–2.209) *	1.929 (1.741–2.136) *	1.402 (1.235–1.592) *	1.437 (1.276–1.618) *
6 or more episodes	2.683 (2.200-3.273) *	2.776 (2.399–3.212) *	1.670 (1.333–2.091) *	1.854 (1.552–2.214) *

*All statistically significant relationships (*p*-value < 0.05)

## Discussion

Nearly one third of the adolescents reported non-use of contraception at first sexual intercourse. Fewer boys than girls reported use of contraception. Having parents with no college/university education, perceived poor family finances, alcohol intoxication, cannabis or other substance use at least one or more times during the last 12 months were positively associated with non-use of contraception at first sexual intercourse for both sexes.

Our findings that a third of adolescents did not use contraception at first sexual intercourse is consistent with a master’s thesis on Norwegian adolescents [8]. However, the percentage is higher than in other studies from Scandinavia, the United States and Croatia, where about a quarter of participants reported not using contraception during their first sexual intercourse. The divergent findings can be attributed to several factors, including the utilization of outdated data and a limited number of respondents. Additionally, cultural, religious, political and social aspects contribute differently to contraceptive practices across various countries [9–12]. It is a surprising finding that so many adolescents did not use contraception during their first sexual intercourse, given that Norwegian adolescents have good access to contraception. Public health nurses and midwives can prescribe contraceptives, which are free for young people under 22 years, and almost all municipalities have their own free youth health centre, which is open after school hours and provides contraception advice and accessible and safe contraception [21]. Adolescents under the age of 16 can receive contraception without parental consent or notification [20]. An explanation for the high number of adolescents not using contraception, despite its good availability, could be that sex education that deals with the use of contraceptives starts too late. In our study, 49% of the adolescents were under the age of 15 when they had their first sexual intercourse. Sex education in Norway is supposed to be age-appropriate and in line with the school curriculum [21], and most schools in Norway provide sex education about contraceptives and their use in 9th grade, the year when most pupils turn 15 years old [27]. Surveys among adolescents show that they feel that sex education starts too late and is insufficient [28, 29], but they also say that it is easy to access contraception and that there is openness in society regarding the use of contraception [30]. The high number of adolescents not using contraception at first sexual intercourse is concerning, especially considering the increase in pregnancy terminations and sexually transmitted diseases among adolescents in Norway in recent years [1, 2]. In terms of sexual health promotion and prevention for adolescents, a key question is whether contraception education should be provided at an earlier stage and be more comprehensive. It is therefore important to provide adequate information about the low-threshold services at youth health centres, where adolescents receive contraception counselling, testing and information about sexually transmitted diseases, as well as advice on pregnancy termination.

Fewer boys than girls reported using contraception at first sexual intercourse, which is consistent with previous findings [8, 12, 31]. The questionnaire used in the study did not provide a clear definition of contraception, which may have resulted in varying interpretations among boys and girls. Thus, not everyone who had used contraception might have reported it, as boys may have only considered condoms as contraception and girls may have only thought of hormonal contraception. This may have inflated the numbers of respondents who reported not using contraception. However, a condom is the most commonly used contraceptive during first sexual intercourse, and a study found that the most frequently reported reason for not using a condom during first intercourse was that it was not available [9]. A possible reason for fewer boys using contraception could be that boys visit youth health centres to a lesser extent than girls [30]. To reach out to boys more effectively, it is important for them to feel that the services are tailored to their needs [21]. Other reasons for not using contraception reported by adolescents in other studies include disadvantages associated with contraception such as concerns about hormones, safety and side effects, as well as the adolescents’ social context [32]. Norwegian girls report that factors inhibiting the use of contraception include limited knowledge about side effects and insertion methods of implants and intrauterine devices, as well as personal traits such as being forgetful and thus forgetting to take the pill [29]. A systematic review of reasons for not using hormonal contraception found the following explanations: concerns about physical side effects, altered mental health, negative impact on sexuality, worries about future fertility, unnaturalness, concerns about menstruation, fear and anxiety, and delegitimization of the side effects of hormonal contraceptives [33]. Health professionals have noticed increasing scepticism of the use of hormonal contraception in recent years, which they believe may be linked to stories of perceived negative side effects of hormonal contraception posted on social media [34].This emphasizes the importance of comprehensive contraception counselling and sex education, as well as accessible youth health services for both boys and girls.

There was an association between having parents without college/university education and perceived family finances and non-use of contraception at first sexual intercourse in both girls and boys. This corresponds well with other studies, where there is generally a connection between lengthy education and financial stability and a healthy lifestyle and good health [35, 36]. A systematic review has found associations between parent-youth sexual communication and youth sexual attitudes and safe sex effectiveness [37]. This highlights the importance of universal preventive measures that reach all adolescents.

We also found an association between non-use of contraception and alcohol intoxication and use of cannabis and other narcotic substances, which is consistent with previous findings [8, 15]. The association was strongest in the group of adolescents who reported having been drunk or having used cannabis or other substances six or more times in the last 12 months, for both sexes. The use of alcohol and drugs can lead to reduced impulse control and thus possibly be a contributing factor to forgetting contraception [6]. A recently published Norwegian study found that both girls and boys reported alcohol and substance use as reasons for not using condoms during first sexual intercourse [9]. This suggests the importance of emphasizing possible reasons for non-use of contraceptives in individual consultations and in universal preventive efforts that reach all adolescents.

### Strengths and limitations of the study

The strength of this study is that Ungdata, with its large sample size, provides a good cross-section of a general population of Norwegian adolescents, as well as representation of respondents from across the country. Data were collected between 2020 and 2022 and thus present an up-to-date description of Norwegian adolescents’ lives. The Ungdata response rate of around 65% of adolescents attending upper secondary school during the same period is relatively good [22–24], but the risk of selection bias must be taken into consideration [38]. The data in Ungdata are based on self-report questionnaires, where questions about alcohol intoxication and use of cannabis and other narcotic substances refer to the past 12 months, which may potentially lead to recall bias that must be considered when interpreting the findings. The adolescents may also have interpreted the questions differently than the researchers intended, which could affect the internal validity of the present study. As a further limitation of the study, it should be mentioned that there is no separate question in the questionnaire about contraception use during same-sex intercourse. This may have resulted in adolescents who engage in same-sex intercourse not answering the question, thereby introducing selection bias in the study. The use of cannabis and other narcotic substances is illegal in Norway and may have resulted in underreporting. Ungdata is a cross-sectional study, and no conclusions can be drawn about causal relationships. The cross-sectional design poses problems with the questions on alcohol intoxication and substance use in the last 12 months and the use of contraception at first sexual intercourse, as it does not directly ask about alcohol intoxication or substance use at the time of first sexual intercourse. Logistic regression was used because it provided a way to measure how strongly non-use of contraception at first sexual intercourse was related to each independent variable. This helped to identify the most influential factors affecting contraception use and to control for other variables that could confound the results. However, the regression analysis used can be sensitive to outliers, which may be the case in our data.

## Conclusion

Many adolescents did not use contraception during their first sexual intercourse. Fewer boys than girls reported the use of contraception at sexual debut.

Having parents without college/university education, previous alcohol intoxication and use of cannabis or other narcotic substances increased the likelihood of not using contraception at first sexual intercourse. This highlights the importance of individual and universal preventive efforts, where sexual education is introduced at an earlier stage, and describes in detail the consequences of not using contraception, in order to prevent unplanned pregnancies and sexually transmitted infections.

## Data Availability

The data cannot be shared by the authors but are available from the Norwegian Agency for Shared Services in Education and Research on request: https://sikt.no/en/tjenester/finn-data/survey-bank.
